# Efficacy of a water flosser compared to an interdental brush on gingival bleeding and gingival abrasion: A 4 week randomized controlled trial

**DOI:** 10.1111/idh.12817

**Published:** 2024-07-12

**Authors:** Deborah Mancinelli‐Lyle, Fridus (G. A.) Van der Weijden, Dagmar E. Slot

**Affiliations:** ^1^ Department of Periodontology Academic Centre for Dentistry Amsterdam (ACTA) A Joint Venture between the Faculty of Dentistry of the University of Amsterdam and the Faculty of Dentistry of the Vrije Universiteit Amsterdam LA Amsterdam The Netherlands

**Keywords:** gingival bleeding, gingivitis, gingival abrasion, interdental brush, oral irrigator, water flosser

## Abstract

**Aim:**

To determine the efficacy of a water flosser (WF) compared to an interdental brush (IDB) in reducing gingival inflammation. Additionally, the products were compared on the incidence of gingival abrasion.

**Methods:**

Young adults with moderate gingivitis and ≥4 accessible interdental spaces by IDB in each quadrant were selected for this study. Participants were randomly assigned a WF or an IDB as an adjunct to manual toothbrushing. Clinical signs of inflammation were measured in two randomly assigned contralateral quadrants by bleeding on pocket probing (BOPP) or bleeding on marginal probing (BOMP). Gingival Abrasion Score (GAS) was assessed per quadrant. Data was recorded at the baseline, 2 weeks and 4 weeks.

**Results:**

Both groups WF (*n* = 40) and IDB (*n* = 38) showed a significant reduction (*p* = 0.000) in BOMP and BOPP from the baseline to 4 weeks for all sites and the interdental sites only. At 4 weeks the WF group compared to the IDB group showed significantly lower BOPP (*p* = 0.030) and BOMP scores (*p* = 0.003) for all sites. For the interdental sites WF showed compared to IDB for BOMP significant (*p* = 0.019) lower values but not for BOPP (*p* = 0.219). There were no differences between the groups for GAS at any time point.

**Conclusion:**

In patients with moderate gingivitis, after 4 weeks use the WF is more effective than the IDB in obtaining marginal gingival health.

## 1 INTRODUCTION

Daily oral hygiene is important for the preservation of oral health. The primary outcome of any oral hygiene regimen is to maintain a microbial ecosystem that prevents the proliferation of pathogenic bacteria from causing dysbiosis leading to gingival inflammation. Toothbrushing and interdental cleaning are considered the mainstays for achieving adequate plaque control every 24 h to prevent the onset of gingival inflammation.^1^ Toothbrushing alone is only in part effective, as it cannot reach the interdental and subgingival regions around the tooth. Interdental devices are designed to reach these areas and supplement toothbrushing to access all regions susceptible to plaque accumulation effectively. Together these two devices help individuals control the build‐up of dental plaque and help to maintain microbiological symbiosis.^1^, ^2^

A meta‐review that focused on the efficacy of various interdental devices for mechanical plaque control in managing gingivitis concludes that interdental cleaning with an interdental brush (IDB) is the most effective method for reducing interdental plaque.^2^ This synthesis was based on data emerging from previously published systematic reviews which provided a moderate level of evidence for recommending interdental brushing in addition to toothbrushing.^3^, ^4^, ^5^ This same meta‐review also concluded that there is a weak level of evidence for the adjuvant use of the water flosser (WF) to daily toothbrushing with respect to gingivitis scores.^6^ A more recent systematic review not included in the meta‐review reported low certainty that string floss or IDBs and toothbrushing may reduce gingivitis, plaque, or both better than toothbrushing alone, and IDBs may be more effective than floss. However, the effect size may be clinically insignificant. This review also reported limited and inconsistent evidence for wood sticks and oral irrigators.^7^

A recent Bayesian Network Meta‐Analysis showed that except for toothpicks, all interdental oral hygiene aids were more effective at reducing gingival index scores than control. IDBs, as well as water flosser, ranked high with respect to the reduction of gingival bleeding scores.^8^ In a recent multi‐outcome secondary analysis with equal weight on the Gingival Index and bleeding on probing, the water flosser and IDB remained ranked as the 2 best interdental cleaning devices.^9^

At present two small pilot studies provide a direct comparison of the WF to IDBs in single‐use model^10^ and a 2‐week follow‐up evaluation.^11^ This present four‐week clinical trial was therefore designed to assess the effect of a WF as compared to an IDB, particularly evaluating bleeding and abrasion scores in systemically healthy gingivitis participants.

## 2 MATERIALS AND METHODS

The recommendations for strengthening reporting as presented in the guidelines Consolidated Standards of Reporting Trials (CONSORT)^12^ and Template for Intervention Description and Replication (TIDieR)^13^ were followed. The protocol was independently reviewed and approved by the Medical Ethics Committee of the Academic Medical Centre in Amsterdam (NL58265.018.16 METC 2016_175) and registered at the Dutch Trial Register (NTR6081).

### 2.1 Ethical aspects

This study was conducted following Good Clinical Practice (CPMP/ICH/135/95) guidelines, in agreement with the ethical principles of the Declaration of Helsinki 1965 (Helsinki, Finland) and revised in 2013 (Fortaleza, Brazil) in accordance with the Medical Research Involving Human Participants Act (WMO), and applicable local regulations.^14^

The study was conducted from October – December 2016 at the Department of Periodontology at the Academic Centre for Dentistry Amsterdam, the Netherlands. A database that contained individuals from universities in and around Amsterdam who agreed to be listed as possible participants in a clinical research study was used for recruitment. Additionally, flyers, posters and advertisements were used to attract additional participants.

An information letter was sent by e‐mail to inform the participants about the background of the study, objectives, duration, and their involvement. After consideration, individuals who wished to participate were scheduled for a screening visit. Participants were asked to read and sign the informed consent and a medical history form before screening for eligibility. To ensure privacy and protect anonymity, each participant received a unique trial number. The participants were financially compensated for volunteering their time in this study and for their travelling costs. The sponsor monitored the study.

### 2.2 Study design

The study was planned as a single‐blind, single‐centre, parallel, four‐week clinical trial. Random allocation was based on a randomization scheme devised by www.random.org. No stratification was applied. The study coordinator and principal investigator (GAW) was responsible for allocation concealment. The examiners were blinded to the treatment randomizations, and records of earlier examinations were not available at re‐examination. Professional instruction took place in an area separate from the examiners. The randomization code was kept in a sealed envelope that was not accessible by the examiners. Participants were strongly advised not to reveal their product assignment to the examiners.

### 2.3 Study population

Systemically healthy, non‐smoking participants without any professional dental relationship were enrolled. Subjects were included if they were between 18 and 65 years, brushed with their right hand, did not take medications except for birth control pills, and were not pregnant or breastfeeding (self‐reported). Those who required antibiotic prophylaxis or participated in another clinical study within 30 days prior that could interfere with the outcomes of this study were excluded. Subjects were not enrolled if they had orthodontic banding, except for lingual retention wire, crowns, bridges, implant‐supported restoration, removable partial denture or night guard, overt dental caries, oral and/or peri‐oral piercings, or oral lesions. Inclusion in the study was also predicated on a minimum of 20 natural teeth with 5 evaluable in each quadrant, moderate to severe gingivitis defined as ≥50% BOPP and no pockets deeper than 5 mm, not including the distal of the last molar. Antibiotic use in the 3 months prior to the study start date and use of any interdental device as part of regular daily oral care were reasons for exclusion. Subjects also had to have buccal accessible interdental spaces to insert the IDB. When inserted from the buccal aspect, the IDB had to fit between at least 4 spaces per quadrant. Two of the 4 spaces had to involve the molar area. The subjects agreed to brush with a manual toothbrush for the study and between 2 and 3 h before clinical measurements. Also, to refrain from rinsing with an antiseptic mouthwash, using any other interdental devices, or excessive gum use (using >3 pieces of chewing gum daily).

### 2.4 Study products

Products were dispensed in labelled non‐transparent paper bags containing study number, emergency phone number, distributor name/address, appropriate caution statements, instruction leaflets, and information regarding study visits and protocol (Appendix S1). All participants were provided a standard manual toothbrush (IQ Lactona, Bergen op Zoom, the Netherlands, flat trim soft: 42 tufts, 9.5 mm polished, end‐rounded soft bristles) and a standard fluoride toothpaste (HEMA Amsterdam, The Netherlands, regular sodium fluoride dentifrice). They were provided with written instructions concerning the Bass toothbrushing technique and told to use the toothbrush in combination with the toothpaste twice a day (Appendix S2).^15^ Participants were randomly assigned to either the WF test group or the interdental brush (IDB) control group. Both products were provided and individually instructed, followed by supervised study product use. This was performed by a dental hygienist (DES), who was familiar with the products and their use and did not participate in the clinical examinations. The participants were provided a diary to record the use of their products and any deviations from the protocol. This was reviewed at each visit and recorded by the practical study coordinator (NLHH).

#### 2.4.1 The WF group

Participants in the test group received the Waterpik® Water Flosser (Water Pik, Inc., Fort Collins, CO, USA), a power‐driven device with a reservoir, pressure control, and a pulsating stream of water (Appendix S3). Participants filled the reservoir with 500 mL of lukewarm water and used the Classic Tip designed for generalized interdental cleaning. The WF was used once a day following written instructions by directing the tip at a 90‐degree angle to the long axis of the tooth at the gingival margin and following a pattern around the mouth, pausing briefly at the interproximal site on the buccal and lingual aspect of the arch (Appendix S4).^14^ The pressure dial was set to 8 for the duration of the study.

#### 2.4.2 Interdental brush group

Participants in the control group also received an interdental oral hygiene device; the Conical brushes with handle (Interprox premium nano, Dentaid, Barcelona, Spain; brush diameter 1.9 mm, core diameter 0.38 mm). The brush is designed to clean interproximal spaces from 1.0 mm and is recommended for teeth in both the posterior and anterior areas of the mouth. The nylon filaments are held together with a stainless‐steel core wire coated with polyurethane (Appendix S5). The brush is held by the handle, adjusted by bending if necessary to be inserted in a straight line between the teeth. Participants inserted the brush the entire length containing the bristles and moved it in and out with 5 strokes and light pressure. The brush was used from the buccal aspect of the dentition and only used in spaces that could accommodate the brush size. Written instructions were provided (Appendix S6).^15^

### 2.5 Outcome parameters

Participants returned to the clinic and each visit started with a clinical assessment under the same conditions per index by the same trained and experienced examiners (EvdS, SS, PAV).

#### 2.5.1 Bleeding on pocket probing scores (BOPP)^16^ and bleeding on marginal probing (BOMP)^17^, ^18^, ^19^, ^20^

A plastic force‐controlled probe (0.25 N) (Click‐Probe®, KerrHawe, Bioggio, Switzerland) was used to record BOPP at six sites per tooth: disto‐buccal, buccal, mesio‐buccal, disto‐lingual, lingual and mesio‐lingual by examiner EvdS. A WHO approved ball‐ended probe (Ash Probe EN15, Dentsply International, York, PA, USA) was used to assess BOMP. The probe was inserted approximately 2 mm and run gently along the marginal gingiva at an angle of approximately 60° and in contact with the sulcular epithelium by examiner SS. The bleeding score was recorded at six gingival areas of the tooth: disto‐buccal, buccal, mesio‐buccal, disto‐lingual, lingual and mesio‐lingual. Bleeding was recorded within 30 s of probing (Appendix S7). Assessment of BOPP and BOMP was performed on two contralateral randomly assigned quadrants.

#### 2.5.2 Gingival abrasion score (GAS)^21^, ^22^, ^23^, ^24^

An undiluted plaque staining solution (Mira‐2‐Ton, Hager & Werken, Duisburg, Germany) was used to expose non‐visible abrasion on the gingival tissue. The examiner PAV used a saturated cotton swab to paint the staining solution on the gingival tissue. Subsequently, the participant rinsed with a sip of water and the gingival tissue was dried for better visibility. Abrasions were recorded by size and location (Appendix S7).

There were no changes to the planned study design.

### 2.6 Study procedure

**FIGURE 1:**
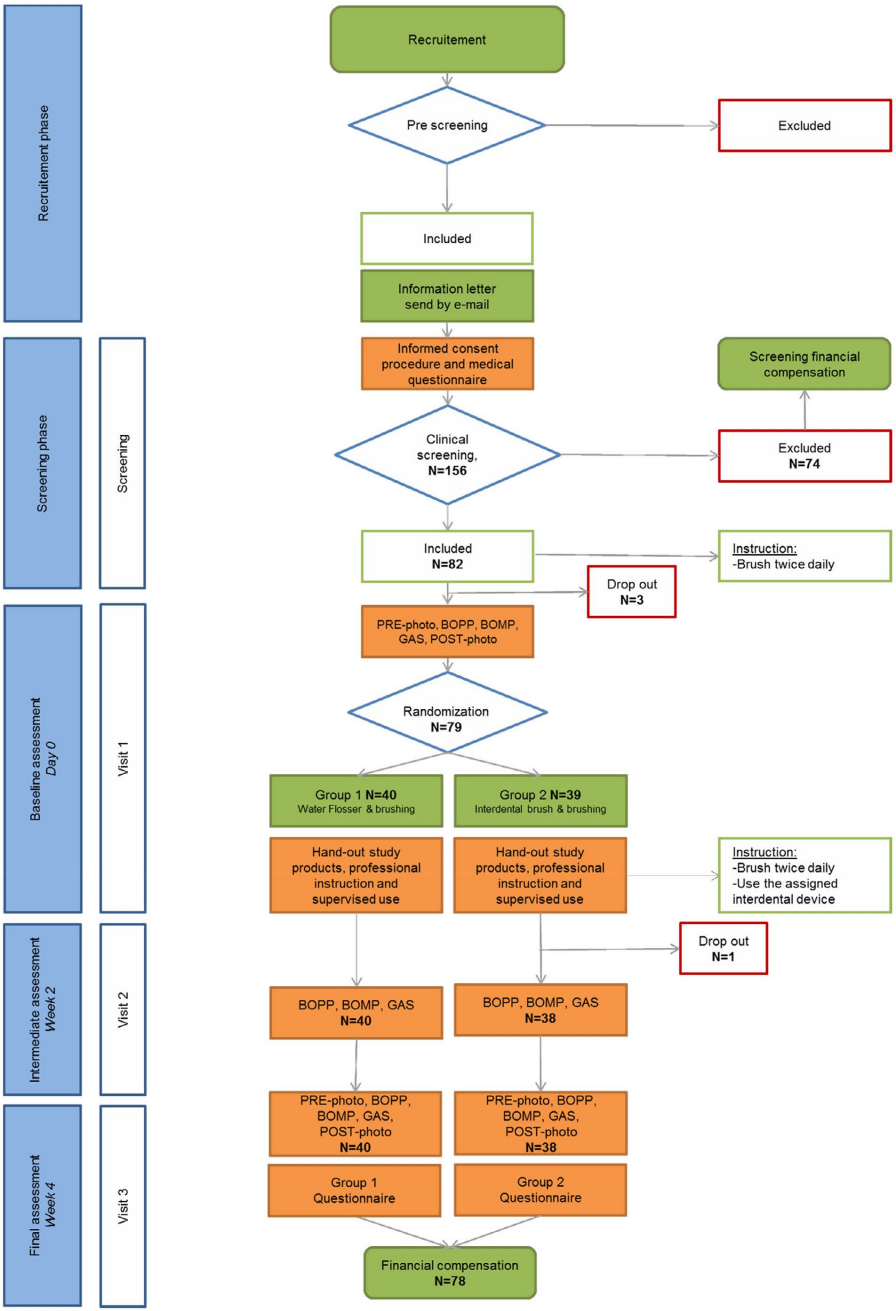
Study flow chart.

Figure 1 shows the flow chart for the passage of the participants through the clinical trial from recruitment to the end of the study. Personal medical history and an intra‐ and extra‐oral examination were conducted at the screening appointment. Inclusion and exclusion criteria were reviewed and an examination was performed to confirm that the volunteer met the clinical inclusion criteria. If a suitable volunteer agreed to participate in the study, they were scheduled for three subsequent visits at the same time on the same day of the week. Data were collected at the baseline, 2 weeks (W2), and 4 weeks (W4) and entered on case report forms (CRF). The study followed the American Dental Association (ADA) guidelines for seal of acceptance categories powered interdental cleaners or WFs and interdental cleaners.^25^, ^26^ Adverse events were reported and followed until they had abated or until a stable situation was reached. Before screening and each visit, a text message was sent to their mobile phone as a reminder for specific protocol aspects, such as brush 2–3 h prior to the appointments, bring the study products and/ or visit the clinic for the appointment.

### 2.7 Power calculation

The ‘a priori’ sample size calculation indicated that 68 participants (34 per group) were needed to provide an 80% probability power to detect a between‐group significant difference of 0.11^27^ assuming a standard deviation [SD] of 0.16 based on a previous studies^28^, ^29^ for mean BOPP at a 0.05 significance level (2‐sided). Accounting for possible drop‐outs the required number of participants was increased by 15%.

### 2.8 Statistical analyses

The statistical analysis was performed blinded to the product assignment using SPSS 23 Software (IBM, Armonk, NY, USA). Study demographics were descriptively analysed. Mean values and standard deviations for each index were calculated for all sites and those sites that were accessible to an interdental brush. Data was tested for normal distribution. All analyses comparing differences between the WF test and IDB positive control group were performed using parametric or non‐ parametric tests where appropriate. Explorative analyses were performed to investigate the origin of the overall differences. Data were then categorized according to upper and lower jaw, tooth types and surfaces, and regions of interest. *P*‐values of *p* < 0.05 was accepted as statistically significant. For the oral soft/hard tissue safety assessment descriptive analysis frequencies were calculated.

## 3 RESULTS

As shown in Figure 1 in total, 156 volunteers were screened, 74 volunteers were excluded and 82 volunteers enrolled based on eligibility criteria, and 78 participants completed the study. Three dropped out prior to visit 1 due to scheduling conflicts, and one participant dropped out between visit 1 and 2, which was unrelated to the assigned study product. The demographics of the study population are presented in Table 1.

**TABLE 1:** Final demographic data.

	WF (*N* = 40)	IDB (*N* = 38)	Overall (*N* = 78)	*p*‐Value
Age (years)				
Mean	22.75	23.32	23.03	0.33*
SD	3.09	2.51	2.81	
Minimum	18	18	18	
Maximum	31	29	31	
Gender				
Male	10	12	22	0.62**
Female	30	26	56	

* *p*‐Value for age from a Wilcoxon rank‐sum test.

** *p*‐Value for gender from a Fisher's exact test.

### 3.1 Gingival bleeding

#### 3.1.1 Bleeding on marginal probing

The WF and IDB groups showed a significant improvement from the baseline BOMP for all sites and interdental sites only at W2 and W4. The WF group was significantly more effective than the IDB group in reducing BOMP for all sites at W2 (*p* = 0.002) and W4 (0.003), with a mean difference of 0.21. Likewise, with a difference of 0.18, the WF group was significantly more effective than the IDB group for reducing BOMP scores for interdental sites at W2 (*p* = 0.003) and W4 (*p* = 0.019) (Table 2a).

**TABLE 2:** The overall means and standard deviations of the scores for gingival inflammation measures – each product used in two contra‐lateral quadrants.

(a) Bleeding on marginal probing					
BOMP	WF, *N* = 40		IDB, *N* = 38		Between groups
	Mean (SD)	*p*‐Value*	Mean (SD)	*p*‐Value*	*p*‐Value**
Baseline (all sites)	0.82 (0.29)		0.95 (0.33)		0.082
2 weeks	0.57 (0.29)	0.000	0.81 (0.37)	0.001	0.002
4 weeks	0.54 (0.27)	0.000	0.75 (0.33)	0.000	0.003
Change baseline‐2 weeks	0.26 (0.31)	0.000	0.14 (0.22)	0.001	0.057
Change baseline‐4 weeks	0.28 (0.33)	0.000	0.19 (0.23)	0.000	0.181
Baseline (Interdental sites)	0.80 (0.34)		0.92 (0.35)		0.130
2 weeks	0.53 (0.29)	0.000	0.77 (0.38)	0.001	0.003
4 weeks	0.52 (0.29)	0.000	0.70 (0.35)	0.000	0.019
Change baseline‐ 2 weeks	0.27 (0.34)	0.000	0.15 (0.25)	0.001	0.087
Change baseline‐ 4 weeks	0.28 (0.37)	0.000	0.22 (0.27)	0.000	0.441

(b) Bleeding on pocket probing					
BOPP	WF, *N* = 40		IDB, *N* = 38		Between groups
	Mean (SD)	*p*‐Value*	Mean (SD)	*p*‐Value	*p*‐Value**
Baseline (all sites)	0.60 (0.10)		0.66 (0.13)		0.030
2 weeks	0.54 (0.13)	0.003	0.58 (0.13)	0.000	0.206
4 weeks	0.50 (0.11)	0.000	0.56 (0.12)	0.000	0.030
Change baseline‐2 weeks	0.06 (0.13)	0.003	0.09 (0.11)	0.000	0.206
Change baseline‐4 weeks	0.10 (0.13)	0.000	0.11 (0.13)	0.000	0.980
Baseline (Interdental sites)	0.66 (0.10)		0.71 (0.14)		0.054
2 weeks	0.60 (0.13)	0.007	0.60 (0.15)	0.000	0.919
4 weeks	0.56 (0.12)	0.000	0.60 (0.14)	0.000	0.219
Change baseline‐2 weeks	0.06 (0.14)	0.007	0.11 (0.11)	0.000	0.084
Change baseline‐4 weeks	0.10 (0.15)	0.000	0.12 (0.15)	0.000	0.000

* Compared to the baseline (paired *t*‐test).

** Between groups (independent *t*‐test).

#### 3.1.2 Bleeding on pocket probing scores

Both the WF and IDB groups showed a significant improvement from the baseline for BOPP for all sites and interdental only sites at W2 and W4 (Table 2b). At 4 weeks, the WF group was significantly more effective at reducing BOPP for all sites than the IDB group (*p* = 0.030). However, the baseline data also showed a significant difference for BOPP for all sites (*p* = 0.030), with a difference (0.06) comparable to that at 4 weeks. Sub‐analysis of interdental sites showed no difference between the groups.

### 3.2 Gingival abrasion

Table 3 shows no differences in overall gingival abrasion scores between the groups at any assessment time point in the study. Sub‐analysis showed this was the same for small lesions or interdental sites (Table 3).

**TABLE 3:** The overall means and standard deviations of the number of sites with gingival abrasion.

All sites	Baseline	2 weeks	4 weeks
WF (*N* = 40)	16.70 (11.69)	21.00 (17.80)	19.90 (11.07)
IDB (*N* = 38)	12.76 (9.39)	16.63 (10.20)	17.18 (9.79)
*p*‐Value**	0.106	0.191	0.256
Small lesions			
WF (*N* = 40)	14.13 (10.10)	17.30 (15.49)	17.35 (9.66)
IDB (*N* = 38)	10.87 (7.92)	14.03 (8.84)	14.87 (8.35)
*p*‐Value**	0.118	0.259	0.230
Interdental area			
WF (*N* = 40)	0.65 (0.83)	1.23 (1.377)	2.85 (2.14)
IDB (*N* = 38)	0.58 (0.95)	1.39 (1.37)	3.34 (2.58)
*p*‐Value**	0.726	0.585	0.362

** Difference between groups independent *t*‐test.

### 3.3 Adverse events/safety

There were six reports of adverse events during the study. One participant with localized interdental inflammation and gingival sensitivity was considered related to the study product interdental brush use. The situation resolved, and the participant continued with the assigned product and the study. The other five events were considered mild and not attributed to the study products. These participants continued in the study, and no action was taken.

## 4 DISCUSSION

This randomized controlled clinical trial was designed based on the ADA protocol guideline for power and manual interdental oral hygiene devices to reduce gingivitis. It was a randomized controlled trial with a parallel design and a duration of 4 weeks. ADA requires that the product is safe to use in the mouth and must comply with ISO standards when applicable.^25^, ^26^ The primary outcome of the present study was the effectiveness of the WF compared to an IDB on the level of naturally occurring (not induced by removal of oral hygiene) gingivitis. Both oral hygiene devices showed a significant reduction in gingival bleeding scores. There was a significant difference in favour of the WF test group for gingival inflammation. Concerning gingival abrasion scores, there were no differences between the sites treated with the WF compared to the IDB.

BOPP has long been considered a meaningful indicator of periodontal inflammation.^30^, ^31^, ^32^ Lang et al.^33^ demonstrated that the lack of bleeding is a suitable indicator of periodontal stability. BOMP measures early inflammation at the gingival margin. Both BOPP and BOMP scores were used for the clinical assessment of gingivitis. As has been reported earlier, the tendency of bleeding on probing can be provoked using different methods.^17^, ^18^ A recent publication reported that probing to the pocket or probing the marginal gingiva results in a measure of gingivitis that is not interchangeable.^28^ In this cross‐sectional study, among 336 participants, BOPP scores were significantly higher than BOMP scores. Subsequently, for the present study, both BOPP and BOMP scores were evaluated, each of which we assessed in two contra‐lateral quadrants.^28^ At the baseline and almost all follow‐up measurements, BOMP scores were higher than BOPP. This could result from the extent of inflammation at the gingival margin and not near the bottom of the periodontal pocket. Another explanation could be the use of a plastic force‐controlled probe adjusted at 0.25 N for BOPP.

Toothbrushing is a mainstay for supragingival plaque removal but is insufficient to access interdental and subgingival areas and proximal surfaces of the teeth.^33^ Another device that can clean these areas seems needed. Currently, there are many devices on the market that claim to meet this need, but the evidence varies by product.^3^ The evidence tends to support interdental brushes as the primary device for interdental cleaning.^34^ There are a few areas to consider along with the information reported in systematic reviews. The brush size has to fit between the teeth without causing any trauma to the interdental papilla. At the time, the WF was systematically reviewed based on the comparison of manual brushing and water flossing compared to manual brushing only.^6^ Seven studies were included in the review from 1971 to 2000. Differences in the devices are worth noting: two studies used devices that have not been on the market for decades, two studies used devices that delivered a multi‐jet, high‐frequency fractionated spurts of water, and the remaining studies used a device that delivered a pulsating stream of water under controlled pressure and pulsation. The studies that used the pulsating stream of water are the same device used in this study and showed benefits in reducing the inflammatory parameters measured, i.e. bleeding and gingivitis scores. The fractionated data included showed no difference.^35^ The review addressed the question posed but did not consider the different mechanisms of action, pressure settings, delivery method, and tip design. Like powered toothbrushes, this is important to accurately evaluate WFs (oral irrigators), and additional systematic reviews are needed to which the present study can contribute.

Comparative research on interdental cleaning aids provides practitioners with information to help recommend the product best suited for patients' needs, preferences, and planned outcomes. A meta‐analysis showed that IDB and WF have the highest probability of being the best choice for the reduction of gingival inflammation. Whereas the probability for wood sticks and dental floss was near zero.^8^, ^36^, ^37^ Randomized controlled trials (RCTs) show that WF is superior to dental floss for reducing gingival inflammation and plaque.^38^, ^39^, ^40^, ^41^ A pilot RCT reported superior outcomes in gingival inflammation scores after 2 weeks for the WF compared to IDB.^10^ The WF has also been compared to the air floss powered interdental device, and reported the WF was significantly more effective for reducing inflammation and plaque.^42^, ^43^, ^44^ All interdental devices in these studies demonstrated efficacy, but the WF repeatedly showed superior benefits for improving gingival health in gingivitis patients.

Regarding recommending self‐care devices, it would be prudent to suggest devices that require the least amount of time to complete the regimen, as this may improve patient adherence and overall oral hygiene outcomes. The time to complete brushing was 2 min, but the time for IDB or water flossing was not recorded. Based on the WF setting (#8) and amount of water (500 mL) it is estimated to be 60 s. The IDB was used in areas in the posterior where it fit interproximally using 5 strokes per site would most likely be less than 60 s. Recording the time for each oral hygiene regimen and patient perceptions is recommended for future studies.

This study supports previous studies’ findings comparing different interdental cleaning methods. The WF was superior to IDB for reducing gingival inflammation. This outcome is a better indicator than plaque removal because it shows a reduction in disease. Only measuring plaque removal does not provide evidence of a concurrent reduction in inflammation and is of limited value when making evidence‐based decisions.

## 5 LIMITATIONS

There are limitations in this study. Only one size of IDB was analysed in select locations where it could fit without trauma. When the interdental papilla recedes, the space increases. The size of the IDB should fit snugly in this interdental space. Therefore, patients need IDBs of various sizes.^45^ The participants could not be blinded to the device distributed for use. The WF is not a commonly used interdental oral hygiene device in the Netherlands, which may have affected behaviour and attitude when using the product. The use of interdental devices was also an uncommon oral hygiene habit in this sample population (Table 1) and, likewise, may have affected both participant's behaviour and attitude. The volunteers were mostly students from the university and, thus not a representative sample of the general population.

Plaque was not measured in this study and could be considered a weakness. However, the primary focus was on the change in bleeding from the baseline to the study endpoint and between groups. Differences in plaque removal are not relevant.

Follow‐up studies should consider including patients with early periodontal disease based on the stage, extent, and progression classification.^37^, ^46^ Further research could include variable sizes of IDBs based on optimal accessibility of the interdental space. The study duration was 4 weeks and sufficient to identify changes in gingival health and followed the ADA guidelines for the seal of acceptance demonstrating efficacy. Whether the level of improvement continues and eventually reaches <10% bleeding, the current criterion for gingival health will need to be addressed in a more extended follow‐up study.^46^ A longer prospective study could also address the issue of compliance and motivation to achieve optimum oral health.

## 6 CONCLUSION

Overall, the WF was more effective than the IDB for reducing gingival inflammation in patients with moderate gingivitis over a 4 week period.

## 7 CLINICAL RELEVANCE

### 7.1 Scientific rationale for the study

Gingivitis is a common oral disease among adults. Self‐performed daily oral hygiene is critical as a primary prevention strategy for periodontitis or a secondary prevention strategy for recurrent periodontitis. Many devices are available on the market to accomplish this goal.

### 7.2 Principal findings

Both devices demonstrated significant reductions in bleeding. The WF was significantly more effective than the IDB for reducing marginal gingival bleeding. There were no differences between the devices for gingival abrasions.

### 7.3 Practical implications

Individuals diagnosed with gingivitis can benefit from using a WF or IDB.

## AUTHOR CONTRIBUTIONS

All authors gave final approval and agreed to be accountable for all aspects of work ensuring integrity and accuracy. DML: contributed to the conception, study design data interpretation and drafted the manuscript. GAW: contributed to the conception, study design, protocol, interpretation of data and critically revised manuscript. DES: contributed to conception, study design, protocol, statistical analysis, data interpretation, and critically revised manuscript.

## FUNDING INFORMATION

The first author, Mancinelli‐Lyle, was an employee of Water Pik, Inc. when the study was conducted. Van der Weijden is the owner of Jardin B.V., which is the former owner of www.ragershop.com (a webshop for various brands of interdental brushes). The study was performed by the Department of Periodontology of ACTA with a commission from ACTA Dental Research B.V. In turn ACTA Research B.V. received financial support from Water Pik, Inc. Fort Collins, CO. USA, Water Pik, Inc. Fort Collins, CO. USA provided the study products. The research team at ACTA of Slot and van der Weijden has previously received either external advisor fees, lecturer fees or research grants from oral health care manufacturers. Those manufacturers included Colgate, Dentaid, GABA, Lactona, Oral‐B, Philips, Procter & Gamble, Sara Lee, Sunstar, TePe,Unilever & Water Pik.

## CONFLICT OF INTEREST STATEMENT

The authors declare that there are no conflicts of interest.

## ETHICS STATEMENT

Ethical approval was given by the Medical Ethics Committee of the Academic Medical Centre. In Amsterdam under number NL58265.018.16 METC 2016_175 and registered at the Dutch Trial Register (NTR6081).

## Supporting information


Data S1:

## Data Availability

The data supporting this study's findings are available from the corresponding author upon reasonable request.
