# Supplementary material for: Efficacy of a water flosser compared to an interdental brush on gingival bleeding and gingival abrasion: A 4 week randomized controlled trial
Source: Int J Dent Hyg. 2024 Jul 12;23(1):176–85. doi: 10.1111/idh.12817 (PMC11717974; doi:10.1111/idh.12817)
Supplement: Supplementary file 1 — Data S1: [file IDH-23-176-s001.pdf]

Supporting information for review and online publication only

Efficacy of an interdental brush compared to a water flosser on gingival bleeding and gingival abrasion: 4 week randomized controlled trial

Authors

Deborah Mancinelli-Lyle

Fridus (G.A.) Van der Weijden

Dagmar E. Slot

Online Appendix S1: Schematic illustration of information provided to the Instruction scheme for participants
Online Appendix S2: Instructions for manual tooth brushing
Online Appendix S3: Water flosser
Online Appendix S4: Instructions for the water flosser
Online Appendix S5: Interdental brush
Online Appendix S6: Instructions for interdental brushes
Online Appendix S7: Criteria for bleeding and gingival abrasion scores

Online Appendix S1: Schematic illustration of information provided to the participants

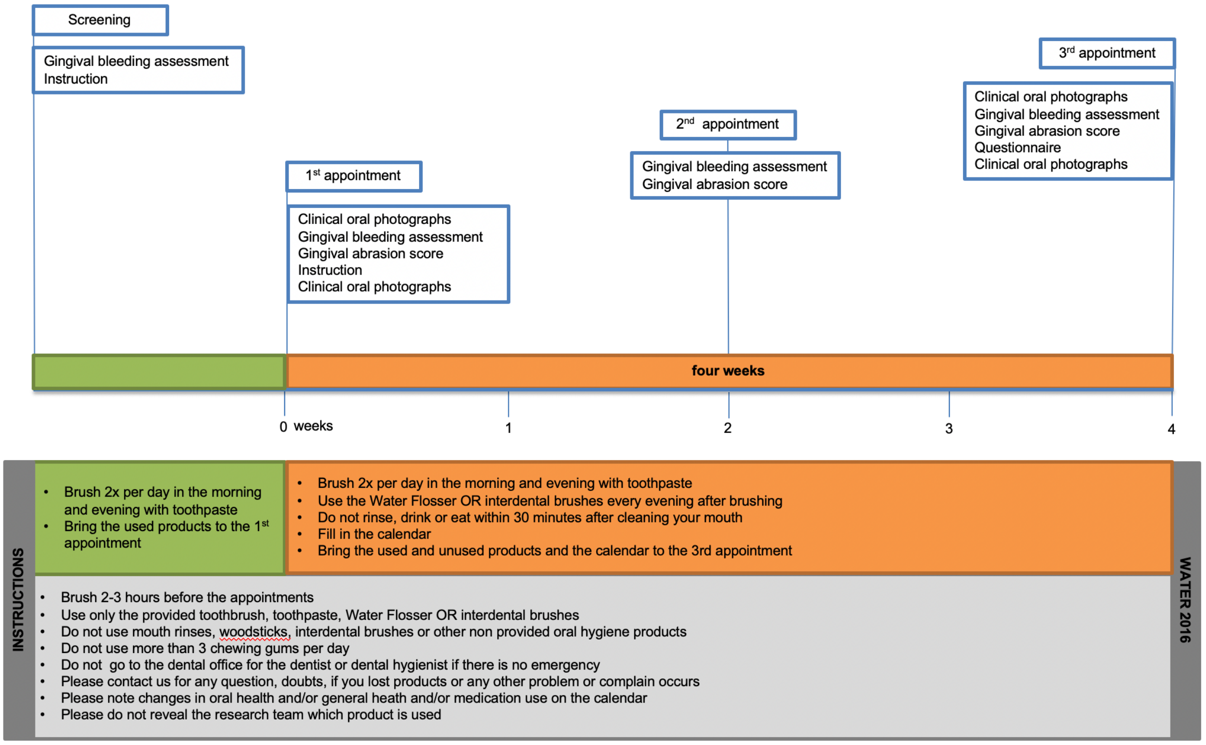

Online Appendix S2: Instructions for the manual toothbrush

Box 36-1 Instructions for manual toothbrushing.

It is of the utmost importance, in addition to using the correct toothpaste and also brushing for at least 2 minutes, to brush the teeth in a set sequence. This technique prevents certain areas from being missed. Areas untouched by the brush will allow plaque to continue to grow. Try to choose a brush with medium or soft bristles and a small head.

Instructions

- Hold the brush firmly and place the bristles at an angle against the edge of your gums (use a 45° angle). Take care to ensure that the bristles are in contact with a small part of the gum margin.
- Place the brush against the molar or tooth at the back of the mouth and make short back-and-forth scrubbing movements. Brush from the back to the front of the mouth, and try to overlap the strokes. Do not brush more than two teeth simultaneously. Always start at the back and work slowly forward.
- Always hold the brush head horizontally when cleaning the outside surfaces of the teeth. It is easier to hold the head vertically when brushing the inside surfaces of the top and bottom teeth.
- Avoid too much pressure and fast movements, and be aware of feeling contact with the gum margin. Also, avoid brushing too vigorously, thereby preventing damage to the gums.

When cleaning the teeth, keep using the same sequence of brushing. For example, brush the inside of the lower left jaw (15 seconds) and then the inside right (15 seconds). Then, brush the left on the outside (15 seconds), followed by the right on the outside (15 seconds). Repeat the same sequence in the upper jaw. Finally, brush the chewing surfaces with small scrubbing movements. Replace the brush when the bristles start to bend or splay.

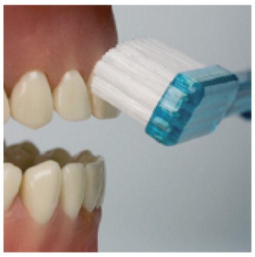

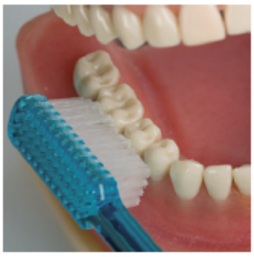

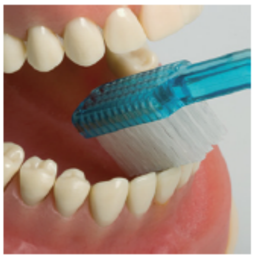

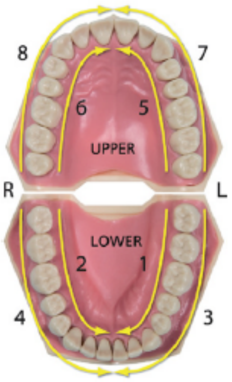

Clinical Periodontology and Implant Dentistry
6th edition
Edited by N.P. Lang & J Lindhe
Wiley Blackwell
ISBN 978-0-470-67248-8
Page 704

Online Appendix S3: Water Flosser

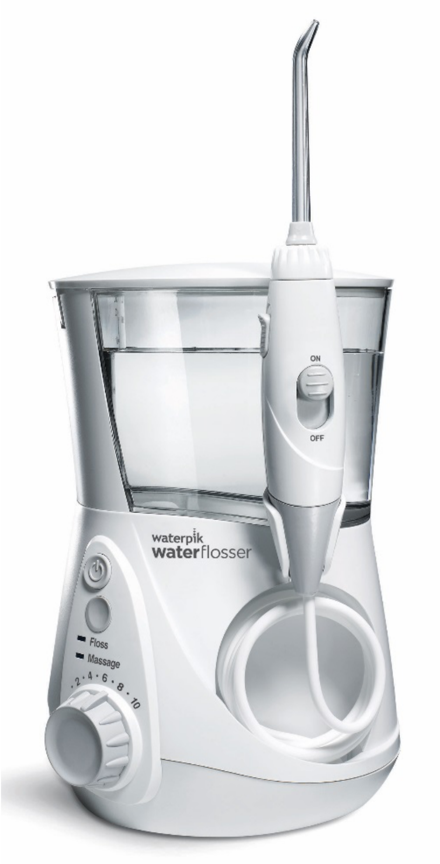

Waterpik® Water Flosser model 660. The device includes 7 tips. The Classic Tip, which was used in the study, is recommended for general use.

Online Appendix S4: Interdental Brush

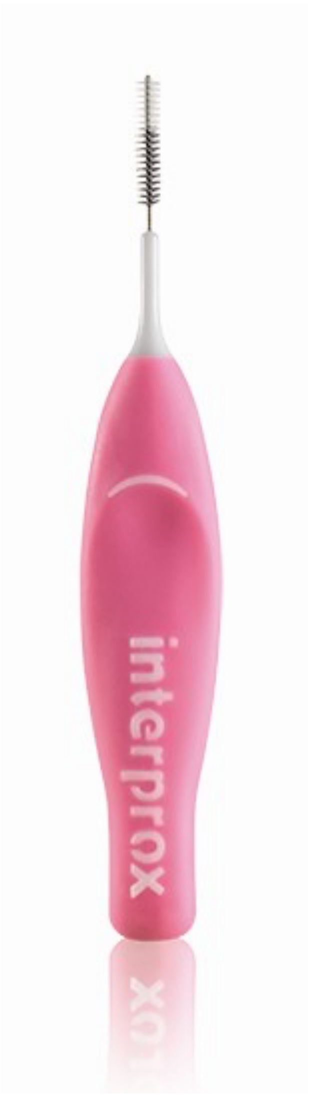

Dentaid nano, brush diameter 1.9 mm, core diameter 0.38 mm

Online Appendix S5:

Instructions for water flosser

Box 36-7 Instructions for use of oral irrigators.

There are various brands of oral irrigators. Before starting to use a product, it is advisable to read the manufacturer's instructions carefully and to be sure you understand how an oral irrigator works.

Instructions

- Fill the water reservoir with lukewarm water, and plug the power cord into the wall outlet. You can use a cup to fill the reservoir. If the unit has removable tips, press the appropriate tip firmly into the irrigator handle. The tip should snap into place because it works under pressure and may shoot away otherwise.
- Test the oral irrigator before use.
- Breathe calmly through your nose. Lean over the sink, and close your lips enough to prevent splashing, while still allowing water to fall from the mouth into the sink.
- Aim the tip just above and toward the gum line at a 90° angle, and press the switch that allows the water to flow.
- *Do not* attempt to watch yourself in the mirror. You will make a mess!
- Starting with the back teeth (where your molars are located), follow the gum line. Take your time to get in between teeth. Continue to work slowly forward until all areas around and between teeth have been cleaned.
- Use the same sequence each time you use the irrigator so that you do not miss any teeth.
- At difficult to reach areas you can adjust the angle of the nozzle, for example while cleaning the brackets of an orthodontic appliance or at root furrows
- Spit out excess water as needed.
- Empty any water remaining in the reservoir after use. Dry thoroughly to avoid bacterial growth. Make sure to unplug the unit before cleaning it.

Irrigating is a technique that relies on your sense of touch. At first, it might take a little longer until you develop a routine and become more comfortable with the oral irrigator. Depending on the power level, you might need to refill the water reservoir. Antiseptics can be added if that has been advised by your dental care professional. If so, a mouth rinse or another antiseptic is added to the water in the reservoir.

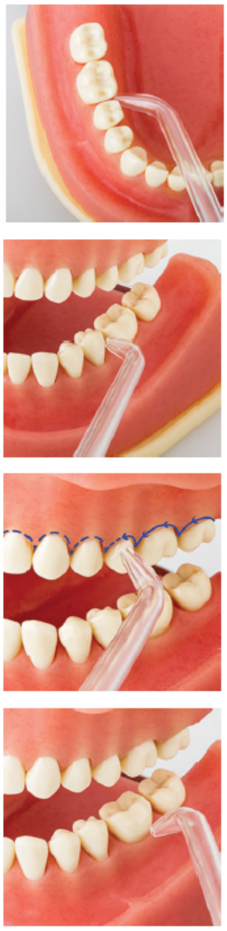

Clinical Periodontology and Implant Dentistry
6th edition
Edited by N.P. Lang & J Lindhe
Wiley Blackwell
ISBN 978-0-470-67248-8
Page 710

Online Appendix S6:

Instructions for interdental brush

Box 36-5 Instructions for use of interproximal brushes.

Interdental brushes can be purchased in a variety of sizes, ranging from small (1.9mm) to very large (14mm). It is important to choose the correct diameter of the bristle part of the brush. The size of the space between the teeth determines the size of the diameter of the bristles on the brush. Dental professionals can precisely identify which sizes you need and also demonstrate their proper use. A brush that is too small will not completely clean the interdental spaces, and a brush that is too large can injure the gums. The wire of an interdental brush must be thin and the bristles as fine and as long as possible. With such dimensions, the interdental brush will fill the entire space between the teeth quite softly and gently. Tooth spacing varies, so it is often necessary to use a different size of brush within one mouth for optimal cleansing. To remove dental plaque effectively, there should be a slight degree of resistance when the brush is moved back and forth between the teeth.

Instructions

- Always use the interdental brush *without* toothpaste.
- Hold the interdental brush just behind the bristles between the thumb and forefinger. Support can be achieved when necessary by placing your other fingers on your chin. From the outer side of the space, push the interdental brush carefully between the teeth, taking care that the brush remains at a right angle to the teeth.
- You may bend the interdental brush slightly to improve accessibility to the posterior interdental spaces.
- Avoid scraping the center (metal spiral part) of the brush against the teeth.
- Slide the brush in and out of the space using the full length of the bristle part of the brush. This action will remove the dental plaque.
- The area of contact between the brush and the teeth can be somewhat increased by using differing angles of insertion.
- Do not push interdental brushes between the teeth with force. Slight pressure of the brush against the gums should be used, as it will allow the bristles to penetrate slightly underneath the gum margin.
- By slightly closing the mouth, it will be easier to manipulate the brush as the tension in the cheeks is lessened. It might also be helpful to bend the brush slightly to ease insertion.
- Cleanse all areas between the teeth where an interdental brush will fit. Rinse interdental brushes thoroughly after use and allow them to dry out. It is often a good idea to combine the use of interdental brushes and woodsticks.

Do not be alarmed if the gums bleed initially. This bleeding does not mean that you have an injury but inflammation, which is caused by concealed, old plaque. This reaction is fairly normal during the first week. Using the interdental brush will soon cure this inflammation, and the bleeding will stop. As the inflammation subsides, the interdental spaces will become slightly larger, and you will most likely need a larger interdental brush. Ask your dental professional.

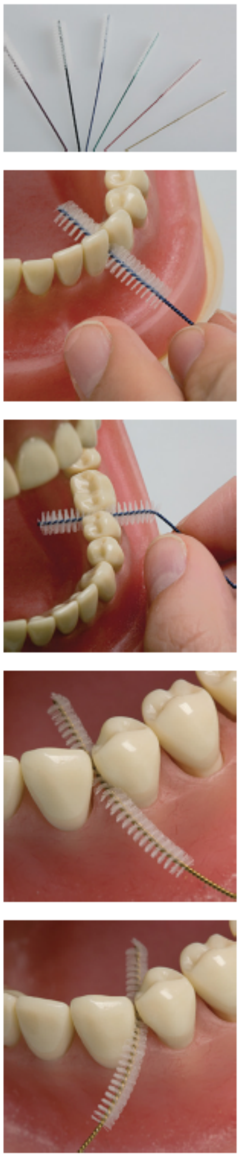

Clinical Periodontology and Implant Dentistry
6th edition
Edited by N.P. Lang & J Lindhe
Wiley Blackwell
ISBN 978-0-470-67248-8
Page 708

Online Appendix S7:

Indices for measurement of bleeding on probing and gingival abrasion

Bleeding on Pocket Probing

0 = No bleeding

1 = Bleeding

Number of Bleeding Sites = % Bleeding site

Total Number of Sites

Bleeding on Marginal Probing

0 = No bleeding

1 = Pin prick bleeding

2 = Excess bleeding

Number of Bleeding PWFnts = % Bleeding pWFnts

Units Probed

Gingival Abrasion Score

Categories

≤ 2.5 mm = small

> 2.5 but ≤ 5 mm = medium

> 5 mm = large

Region

M = molar

P = premolar

I = incisor & canine
